# Composite neuroendocrine carcinoma and squamous cell carcinoma with regional lymph node metastasis: a case report

**DOI:** 10.1186/s13256-018-1775-z

**Published:** 2018-08-24

**Authors:** Shintaro Fujihara, Masahiko Kobayashi, Masako Nishi, Tatsuo Yachida, Akira Yoshitake, Akihiro Deguchi, Atsushi Muraoka, Hideki Kobara, Tsutomu Masaki

**Affiliations:** 1 Department of Gastroenterology, Kagawa Rosai Hospital, 3-3-1, Joutou, Marugame, Kagawa Japan; 2 Department of Surgery, Kagawa Rosai Hospital, Marugame, Kagawa Japan; 3 Department of Gastroenterology and Neurology, Faculty of Medicine, Kagawa University, Miki, Kagawa Japan

**Keywords:** Neuroendocrine cell carcinoma, Esophagus, Squamous cell carcinoma, Endoscopic submucosal dissection, Metastasis

## Abstract

**Background:**

Neuroendocrine cell carcinoma is a rare variant of esophageal carcinoma. The characteristic clinical features and diagnosis of superficial neuroendocrine cell carcinoma remain to be established. We report a rare case of superficial coexistence of neuroendocrine cell carcinoma with squamous cell carcinoma treated by endoscopic submucosal dissection, and regional lymph node metastasis was detected after additional surgical treatment.

**Case presentation:**

A 77-year-old Japanese man with esophageal squamous cell carcinoma received endoscopic submucosal dissection in en-bloc resection. Histopathological findings showed that lymphovascular invasion by the neuroendocrine cell carcinoma component occurred in the deep part of the muscularis mucosa. Regional lymph node metastasis was identified after additional surgical treatment. After surgical treatment, our patient received chemotherapy consisting of etoposide and carboplatin for 3 months. He is alive and shows no sign of disease recurrence 12 months after surgery.

**Conclusions:**

This case report highlights the fact that even if neuroendocrine cell carcinoma is small and limited to superficial, the tumor has the potential for metastasis if lymphovascular invasion by the neuroendocrine cell carcinoma component occurs. In addition, this case indicates the necessity of close follow-up of small neuroendocrine cell carcinoma after treatment.

## Background

Neuroendocrine cell carcinoma (NEC) is rare variant of esophageal carcinoma. NEC is less common than squamous cell carcinoma (SCC) and adenocarcinoma, and it is a relatively rare disease with a reported incidence between 0.4% and 2% [1–3]. The prognosis of NEC of the esophagus is poor because the tumor is often at an advanced stage of disease at diagnosis. Superficial NEC of the esophagus is rare. To the best of our knowledge, only a few superficial NECs of the esophagus have been reported in the English literature [4]. Accordingly, the characteristic endoscopic features and diagnosis of superficial NEC remain to be established.

Endoscopic mucosal resection (EMR) and endoscopic submucosal dissection (ESD) offer non-invasive treatment for esophageal cancer limited to the mucosa and without lymph node metastasis [5]. ESD has a significantly higher curative resection rate and lower local recurrence rate than EMR, particularly in lesions less than 2 cm [6]. However, whether endoscopic treatment is sufficient for disease control for superficial NEC is unclear.

We report a rare case of superficial coexistence of NEC with SCC treated by ESD, and regional lymph node metastasis was detected after additional surgical treatment.

## Case presentation

**Fig. 1:**
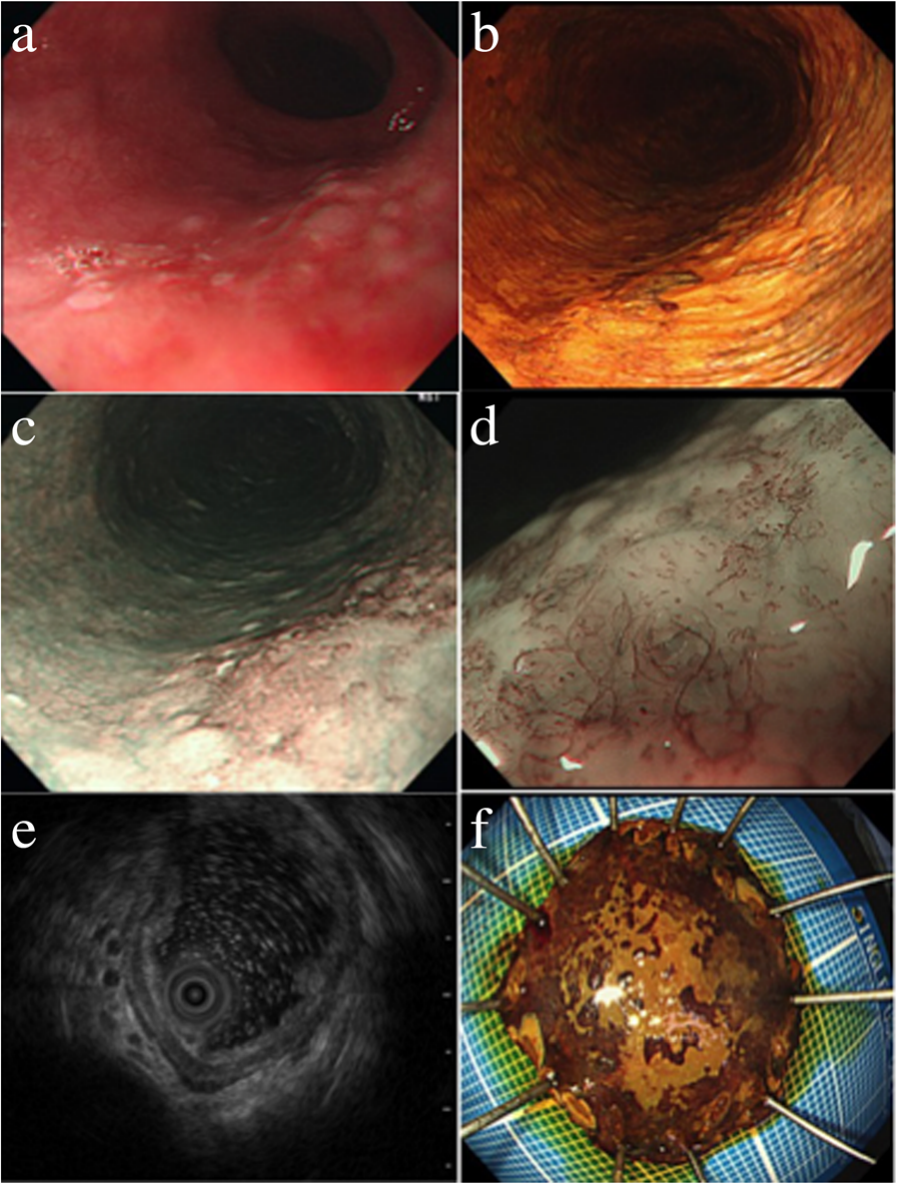
**a** Esophagogastroduodenoscopy shows an irregular, reddish, flat lesion in the posterior wall of the mid-esophagus. **b** Lugol’s iodine chromoendoscopy shows an unstained lesion that is located in the posterior wall of the mid-esophagus. **c** Narrow band imaging endoscopy shows a brownish area. **d** Magnifying endoscopy with narrow band imaging shows a microvascular pattern with irregular, fine, reticular blood vessels near the center of the lesion. **e** Endoscopic ultrasound image of the lesion limited to the mucosa. **f** En bloc resection by endoscopic submucosal dissection

A 77-year-old Japanese man presented to our hospital with esophageal mucosal abnormality in the middle thoracic esophagus. This abnormality was discovered in a barium study for a health checkup. His medical history was significant for primary hypertension. He reported a 50-year history of smoking 8–10 cigarettes per day. There was no family history. Physical and neurological examinations were unremarkable. Esophagogastroduodenoscopy showed 20-mm, reddish, elevated, and flat lesions in the middle thoracic esophagus (Fig. 1a). Narrow band imaging (NBI) endoscopy showed dot-like microvessels in elevated lesions (Fig. 1b, c). However, the microvascular pattern showed irregular, fine, reticular blood vessels, of Japan Esophageal Society (JES) classification type R, near the center of the lesions (Fig. 1d). Magnifying endoscopy with NBI revealed type B1 in elevated area, and type R near the center of the lesion in the JES classification. Endoscopic ultrasound showed that the lesion was localized in the mucosa (Fig. 1e). Therefore, this area of invasion was: cancer limited to the epithelium (EP)/cancer invading into the lamina propria (LPM) to cancer invading into the muscularis mucosa (MM). Biopsies showed SCC of the esophagus. Computed tomography (CT) showed no evidence of lymph node and distant metastases. En bloc resection of the tumor was performed successfully by esophageal ESD without any complications (Fig. 1f).

**Fig. 2:**
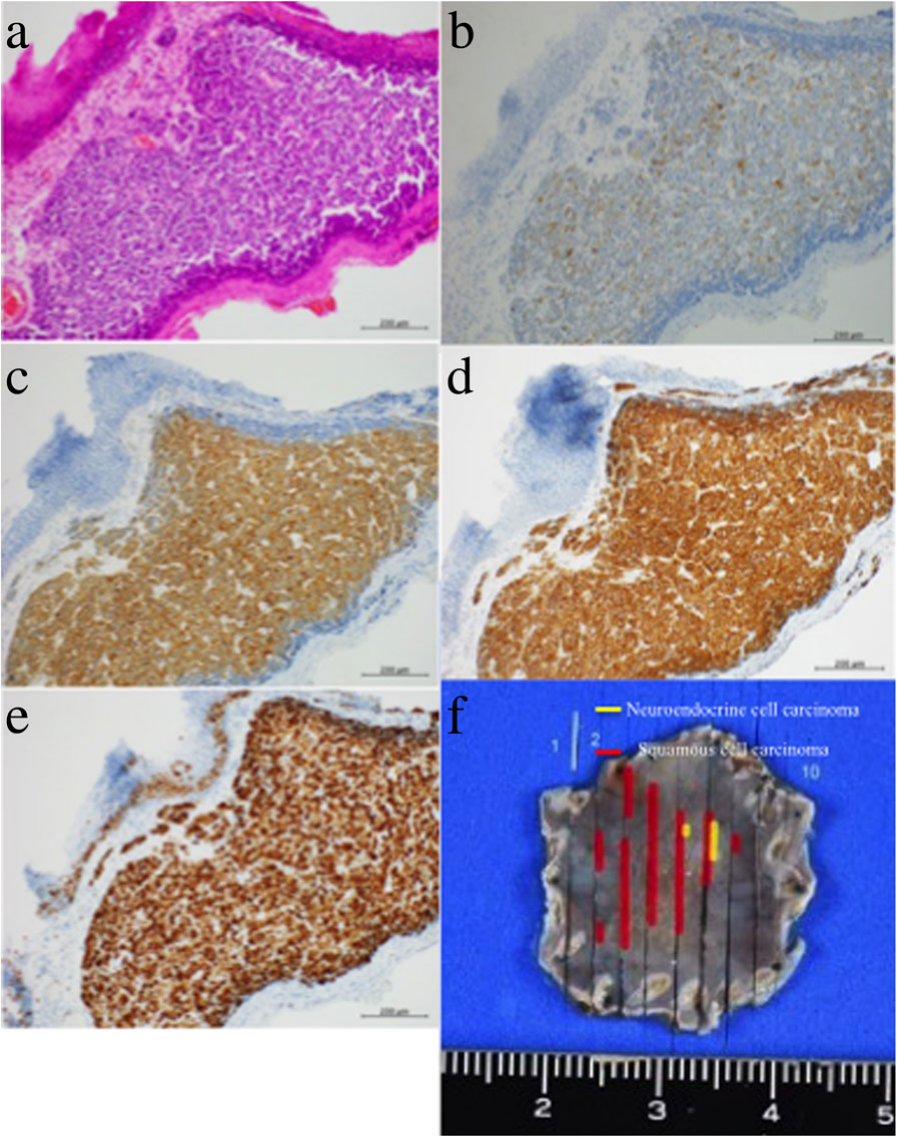
Resected specimen by endoscopic submucosal dissection. This specimen shows neuroendocrine cell carcinoma arranged in a sheet fashion with mixed squamous cell carcinoma. Neuroendocrine cell carcinoma formed a duct and it is surrounded by squamous cell carcinoma. **a** Hematoxylin and eosin staining. Immunohistochemical staining showing: **b** chromogranin A, **c** synaptophysin, **d** CD56, and **e** Ki-67. **f** The fixed resected specimen is mapped by *yellow* and *red lines*. *Red lines* squamous cell carcinoma, *yellow lines* neuroendocrine cell carcinoma

**Fig. 3:**
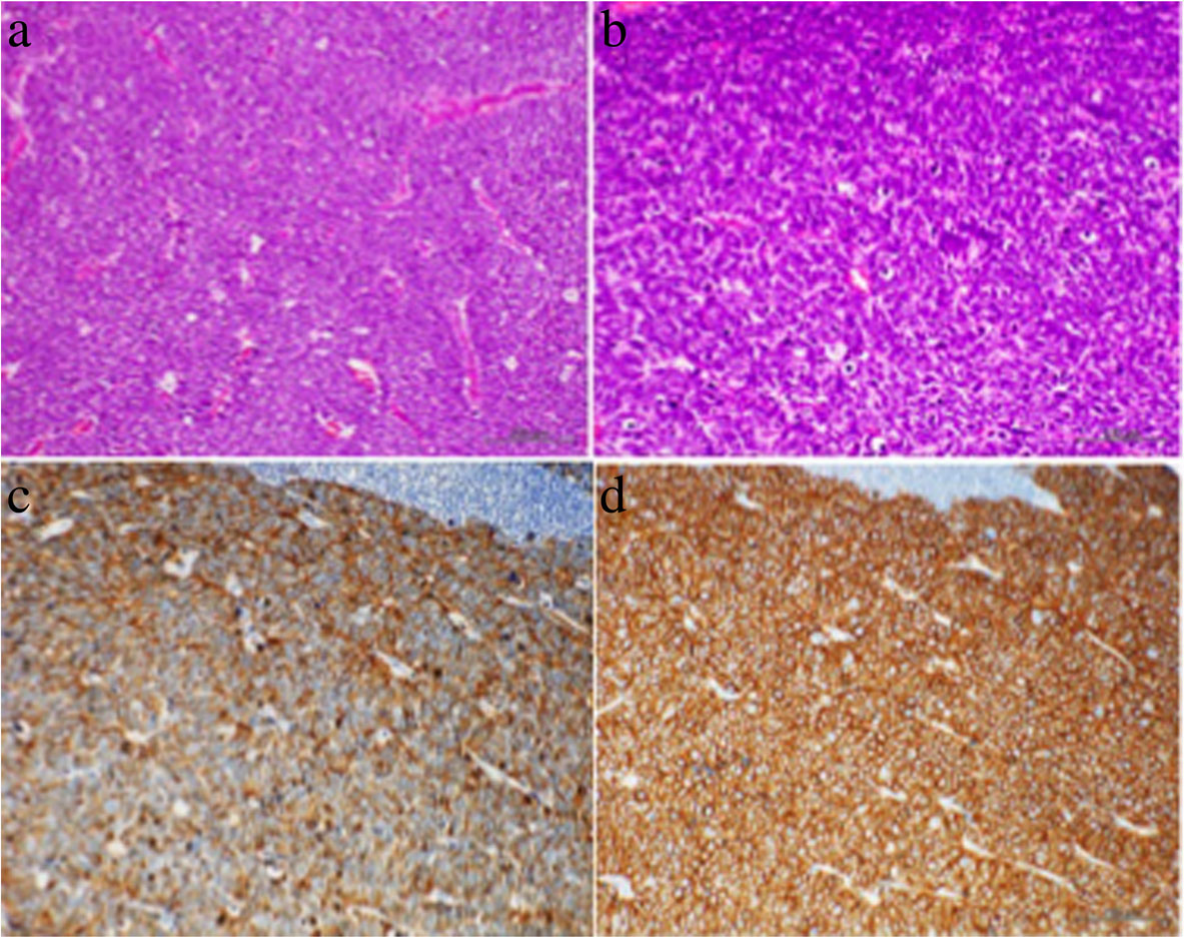
Histological examination of a resected lymph node. **a** Low-power view and **b** high-power view (hematoxylin and eosin staining). Immunohistochemical staining shows: **b** chromogranin A, **c** synaptophysin, and **d** CD56

Histopathological findings showed an admixture of endocrine cell tumor with SCC with an invasion depth into the muscularis mucosa (Fig. 2a). No complications were related to the procedure. Immunohistochemical analysis showed positivity for CD56, chromogranin, and synaptophysin in the NEC component (Fig. 2b–f). Small cell type NEC was arranged in a sheet fashion existing in the center of the tumor, and these were partially surrounded by well-differentiated SCC. Lymphovascular invasion of the NEC component occurred in the deep part of the muscularis mucosa. Our patient underwent additional surgical treatment consisting of video-assisted thoracoscopic esophagectomy, three-field lymph node dissection from the cervix, mediastinum, and abdomen, and gastric conduit construction. Regional lymph node metastasis was identified in 1 of 76 nodes (number 108 lymph node), and the node metastasis stage was pN1. This lymph node contained NEC and no metastasis of SCC (Fig. 3a–d). After surgical treatment, he received chemotherapy consisting of etoposide and carboplatin for 3 months. He is alive and shows no sign of disease recurrence 12 months after surgery.

## Discussion

NEC was formerly called small cell carcinoma. The first description of small cell carcinoma of the esophagus was reported in 1952 by McKeown [7]. The World Health Organization (WHO) definition for NEC includes positive endocrine markers, such as CD56, chromogranin A, and synaptophysin. A Ki-67 or mitotic index of 20% or higher is also necessary for diagnosing NEC. Tumors with less than 20% Ki-67 positivity are diagnosed as a neuroendocrine tumor [8].

NEC is categorized into two morphological types of small cell type and large cell type, and coexistence of SCC and/or adenocarcinoma is also often observed [9]. The small cell type is more aggressive than the large cell type, and it is frequently found in the advanced stage with lymph node and distant metastases [10].

We ascertained two clinically important issues based on the findings in our case. With regard to the first clinical issue, a small neuroendocrine tumor may occur with lymph node metastasis at an early stage, and attention should be paid to metastasis. Neuroendocrine tumors of the esophagus more frequently present in the middle of the esophagus and stain positive for CD56, synaptophysin, and chromogranin A [1, 11]. These tumors are slow growing, but high-grade neuroendocrine tumors of the esophagus have a poor prognosis and a 5-year survival of approximately 25% [1, 11]. The therapeutic strategy for NEC of the esophagus has not been well defined because of the small number of cases [10]. To date, there has only been one case of collision of SCC and NEC of the esophagus treated by ESD [4].

Saddoughi *et al.* performed a retrospective analysis of malignant esophageal cancers including NEC [12]. They found that 1-year survival after surgical resection for rare types of malignant esophageal cancers was 69%, 5-year survival was 43%, and 10-year survival was 37%. Surgical resection for rare types of esophageal malignancies should be considered part of effective treatment. In our case, a resected lymph node contained NEC without an SCC component, and regional lymph node metastasis was identified at the early stage of the NEC. Which patients will benefit from additional therapy at the early stage of NEC has not been well established. Therefore, additional therapy, such as esophagectomy and chemoradiotherapy, is required if histology from endoscopic resection shows a tumor invasion depth into the muscularis mucosa with lymphovascular invasion by NEC components.

Concerning the second clinical issue, the diagnosis of NEC before treatment is difficult. Table 1 summarizes cases of esophageal endocrine cell carcinoma treated by endoscopic resection. Characteristic endoscopic features of NEC include submucosal growth, which is usually covered by normal epithelium with or without an ulcerous lesion in the center [8]. Biopsy sometimes fails to reach any diagnosis of NEC before treatment [4]. In our case, the microvascular pattern showed irregular, fine, reticular blood vessels near the center of the lesion. Vessels with a reticular pattern are defined as plexiform microvessels [13]. This vascular pattern is often found in invasive SCC or a non-SCC type of malignant epithelial neoplasm (for example, basaloid carcinoma, adenosquamous carcinoma, and endocrine tumor) with an infiltrative growth pattern [13, 14]. Therefore, vessels with a reticular pattern are not a specific sign for diagnosis of an early stage of NEC, but physicians have to consider the possibility of non-SCC types of malignancies.

**Table 1:** Cases of superficial esophageal endocrine cell carcinoma treated by endoscopic resection

Case	Author and reference number	Year	Age	Sex	Resection method	Size, mm	Morphology	Depth	Lymphovascular invasion	Treatment after ER	Recurrence after ER, duration after ER (months)	Prognosis, period (months)
1	Takeshita *et al.* [15]	2000	73	F	EMR	5	0–IIa	Muscularis mucosa (M3)	None	Carboplatin + etoposide	None	Alive, no recurrence, 28
2	Ozawa and Wachi [16]	2009	78	M	ESD	7	0–IIa	Shallow submucosa (SM1)	None	CPT-11 + CDDP	Abdominal lymph node metastasis, 9	Alive, 9
3	Kobayashi *et al*. [17]	2011	61	M	ESD	22	0–IIc	Shallow submucosa (SM1)	Yes	5-FU + CDDP, additional surgery, FP + docetaxel, and radiation therapy	Lymph node metastasis around thoracic aorta, 14	Dead, 25
4	Watanabe *et al.* [4]	2014	55	M	ESD	30	0–IIa + IIc	Deep submucosa	Yes	CPT-11 + CDDP	None	Alive, 55
5	Present case	2017	77	M	ESD	20	0–IIa	Muscularis mucosa (M3)	Yes	Carboplatin + etoposide	Regional lymph node metastasis, 1	Alive, 12

*5-FU* 5-fluorouracil, *CDDP* cisplatin, *CPT-11* irinotecan, *EMR* endoscopic mucosal resection, *ER* endoscopic resection, *ESD* endoscopic submucosal dissection, *F* female, *FP* 5-Fluorouracil plus cisplatin, *M* male, *M3* muscularis mucosae, *SM1* submucosa

## Conclusions

In summary, we report a rare case of small superficial NEC showing regional lymph node metastasis. This case report highlights the fact that even if a NEC is small and limited to superficial, the tumor has the potential for metastasis if lymphovascular invasion by the NEC component occurs. In addition, this case indicates the necessity of close follow-up of small NEC after treatment.

## Abbreviations

CT: Computed tomography
EMR: Endoscopic mucosal resection
EP: Cancer limited to the epithelium
ESD: Endoscopic submucosal dissection
JES: Japan Esophageal Society
LPM: Cancer invading into the lamina propria
MM: Cancer invading into the muscularis mucosa
NBI: Narrow band imaging
NEC: Neuroendocrine cell carcinoma
SCC: Squamous cell carcinoma
WHO: World Health Organization
